# Cerebral-Cardiac Syndrome and Diabetes: Cardiac Damage After Ischemic Stroke in Diabetic State

**DOI:** 10.3389/fimmu.2021.737170

**Published:** 2021-08-27

**Authors:** Hong-Bin Lin, Feng-Xian Li, Jin-Yu Zhang, Zhi-Jian You, Shi-Yuan Xu, Wen-Bin Liang, Hong-Fei Zhang

**Affiliations:** ^1^Department of Anesthesiology, Zhujiang Hospital of Southern Medical University, Guangzhou, China; ^2^State Key Laboratory of Ophthalmology, Zhongshan Ophthalmic Center, Sun Yat-sen University, Guangzhou, China; ^3^Guangxi Health Commission Key Laboratory of Clinical Biotechnology, Liuzhou People’s Hospital, Liuzhou, China; ^4^University of Ottawa Heart Institute and Department of Cellular and Molecular Medicine, University of Ottawa, Ottawa, ON, Canada

**Keywords:** cerebral-cardiac syndrome, ischemic stroke, cardiac damage, NLRP3 inflammasome, diabetes mellitus

## Abstract

Cerebral-cardiac syndrome (CCS) refers to cardiac dysfunction following varying brain injuries. Ischemic stroke is strongly evidenced to induce CCS characterizing as arrhythmia, myocardial damage, and heart failure. CCS is attributed to be the second leading cause of death in the post-stroke stage; however, the responsible mechanisms are obscure. Studies indicated the possible mechanisms including insular cortex injury, autonomic imbalance, catecholamine surge, immune response, and systemic inflammation. Of note, the characteristics of the stroke population reveal a common comorbidity with diabetes. The close and causative correlation of diabetes and stroke directs the involvement of diabetes in CCS. Nevertheless, the role of diabetes and its corresponding molecular mechanisms in CCS have not been clarified. Here we conclude the features of CCS and the potential role of diabetes in CCS. Diabetes drives establish a “primed” inflammatory microenvironment and further induces severe systemic inflammation after stroke. The boosted inflammation is suspected to provoke cardiac pathological changes and hence exacerbate CCS. Importantly, as the key element of inflammation, NOD-like receptor pyrin domain containing 3 (NLRP3) inflammasome is indicated to play an important role in diabetes, stroke, and the sequential CCS. Overall, we characterize the corresponding role of diabetes in CCS and speculate a link of NLRP3 inflammasome between them.

## Introduction

Cerebral-cardiac syndrome (CCS) is an interplay between the brain and the heart, which is also known as neurocardiogenic syndrome. Various brain injuries such as ischemic stroke (IS), intracerebral hemorrhage, subarachnoid hemorrhage, traumatic brain injury, and stress are evidenced to cause cardiac injuries or to exacerbate preexisting heart disease, which manifests as arrhythmia, various myocardial damages, heart failure, and myocardial infarction (1–5). CCS can be found after brain injury even without primary heart disease (6). However, it is more likely to occur in the context of cardiovascular and cerebrovascular diseases such as diabetes, hypertension, and obesity (7). IS is a common brain injury, and the consequence of cardiac complications contributes to a higher risk of death in the post-stroke stage (6). In the acute stage following IS, arrhythmias are the most common cardiac complications, while in the chronic stage, cardiac dysfunction, heart failure, myocardial fibrosis, and hypertrophy can also be detected (8–10). Of note, diabetes not only drives the incidence of cardiovascular and cerebrovascular comorbidity but also leads to poor prognosis afterward. However, the key molecular mechanisms and responsible pathways of the interaction of diabetes on CCS are unclear. Inflammation is an important trigger for both diabetes and CCS (11, 12), and NOD-like receptor pyrin domain containing 3 (NLRP3) inflammasome is the crucial element to promote the maturation and release of IL-1β and IL-18 (13). Recent studies have indicated the role of NLRP3 inflammasome in diabetes and diabetic CCS (14, 15). Therefore, the responsible pathway of NLRP3 inflammasome activation and subsequent effects on diabetic CCS need further exploration.

CCS has now gained more attention due to the emerging impact of stroke on cardiac pathophysiological comorbidity. To further dissect the corresponding mechanisms, we herein review and summarize its clinical features, pathophysiological process, and the underlying molecular crosstalk. Furthermore, we summarize the special role and explain the underlying molecular mechanisms of diabetes in CCS. Lastly, we speculate a reasonable link of NLRP3 inflammasome between diabetes and CCS.

## Cerebral-Cardiac Syndrome in Ischemic Stroke

### Clinical Features

The prevalence of patients who died of cardiac complication after IS has been estimated to be about 4%–19% (16–18). Positive correlation has been indicated between cardiac complications and the severity of IS (19). Substantial evidence shows that IS provokes cardiac arrhythmias such as atrial fibrillation, ventricular ectopy, atrioventricular conduction disturbance, or nonsustained ventricular tachycardia (20). Most of the arrhythmias occur in the acute phase, with 93.9% onset in the first day after stroke (21). The most common type is atrial fibrillation, with an incidence rate of 10% within 24 h (22) and 68.7% within 72 h of admission (8). Second- or third-degree atrioventricular conduction block, focal atrial tachycardia, and supraventricular tachycardia are also present. Preexisting cardiac disease is the major cause of newly diagnosed atrial fibrillation (23). Abnormal electrocardiograph (ECG) changes have also been reported in 65%–69% IS patients, including prolonged QT intervals (26%–36%), ST depression or elevation (24.5%–50%), and T wave inversion (17.8%) (24–26). A prolonged QT interval has positive correlation with sudden cardiac death (27), while ST-segment changes predict early mortality (25). Stroke patients benefit from prolonged ECG monitoring, which is effective in detecting atrial fibrillation, particularly for those with elevated hs-cTnT levels and insular ischemia (28). However, it should be noted that more than two-thirds of patients did not receive 24-h Holter monitoring within 30 days of stroke or transient ischemic attack, and only less than 1% patient received prolonged 48-h ECG monitoring (29).

The incidence of several types of myocardial damage increased after IS. It was reported that 88% of patients with right insular cortex ischemia suffered from myocardial injury in the weeks after stroke (30). Kolin et al. found that focal transmural myocardial damage was significantly increased in stroke patients (62%) (31). Myocardial infarction has been reported in 2.2%–4.9% of the stroke population (32, 33). Of note, for those without prior coronary artery disease, the incidence of myocardial infarction reaches 3.5% after IS (34). Besides, studies have reported that different myocardial enzymes elevated after IS, including cardiac troponin I (cTnI) [20.6% (35)], cardiac troponin T (cTnT) [9.6% (24)], high-sensitivity cardiac troponin T (hs-cTnT) [22.8% (36)], and creatine kinase-MB (CK-MB) [34.4% (37)]. The increased myocardial enzymes, especially of hs-cTnT, are reliable indicators of myocardial damage and are useful in predicting poor outcomes after stroke (38). IS also induces cardiac dysfunction after myocardial damage. A total of 11.6% patients have been suffering from left ventricular systolic dysfunction (LVSD) after IS (39). Neurogenic stunned myocardium can also be found after IS and manifests as reversible LVSD (40). In addition, a previous study has suggested that 91.3% of IS patients suffer from takotsubo cardiomyopathy, which is characterized by LVSD and abnormalities in myocardial enzymes and ECG (41). A study has shown that 17% of patients were diagnosed with decompensated heart failure after IS (42). Of note, impaired cardiac function can be a predictor of poor prognosis (39). IS patients with cardiac dysfunction have higher mortality and prolonged hospitalization than those without cardiac complications (43).

Overall, the clinical features of CCS include arrhythmia, ECG changes, various myocardial damages, and heart failure. We herein summarize the multiple manifestations of clinical cardiac damage and the correlated incidence after IS (**Table 1**). As the clinical features of CCS are documented to predict poor prognosis after stroke, it is possible to improve the prognosis and decrease the mortality of stroke by enhancing the diagnosis and initiating treatment for cardiac complications.

**Table 1:** Symptoms and incidence of cardiac complications after IS.

Symptom	Type	Incidence
ECG changes	Atrial fibrillation	10.0% (within 24 h) (22), 17.2% (within 72 h) (8)
	Focal atrial tachycardia	2.9% (within 72 h) (8)
	Second- or third-degree atrioventricular conduction blocks	2.2% (within 72 h) (8)
	Supraventricular tachycardia	2.0% (within 72 h) (8)
	Unidentified	29.5% (within 48 h) (44), 25.0% (within 72 h) (8), 2.0% (within 4 y) (45), 31.0% (46)
	QT prolongation	36.0% (24), 26.0% (25)
	ST changes	24.5% (24), 41.0% (25)
	T wave inversion	17.8% (24), 50.0% (25)
	Ischemia-like ECG changes	64.5% (25)
Myocardial damage	Myocardial injury	88.0% (30)
	cTnI elevate	20.6% (35)
	cTnT elevate	9.6% (24), 10.9% (47)
	hs-cTnT elevate	54.4% (48)
	CK-MB elevate	34.4% (37)
	Myocardial infarction	3.5% (34), 4.9% (32)
Heart failure	Decompensated heart failure	17.0% (10)

### Pathophysiological Mechanisms

**Figure 1:**
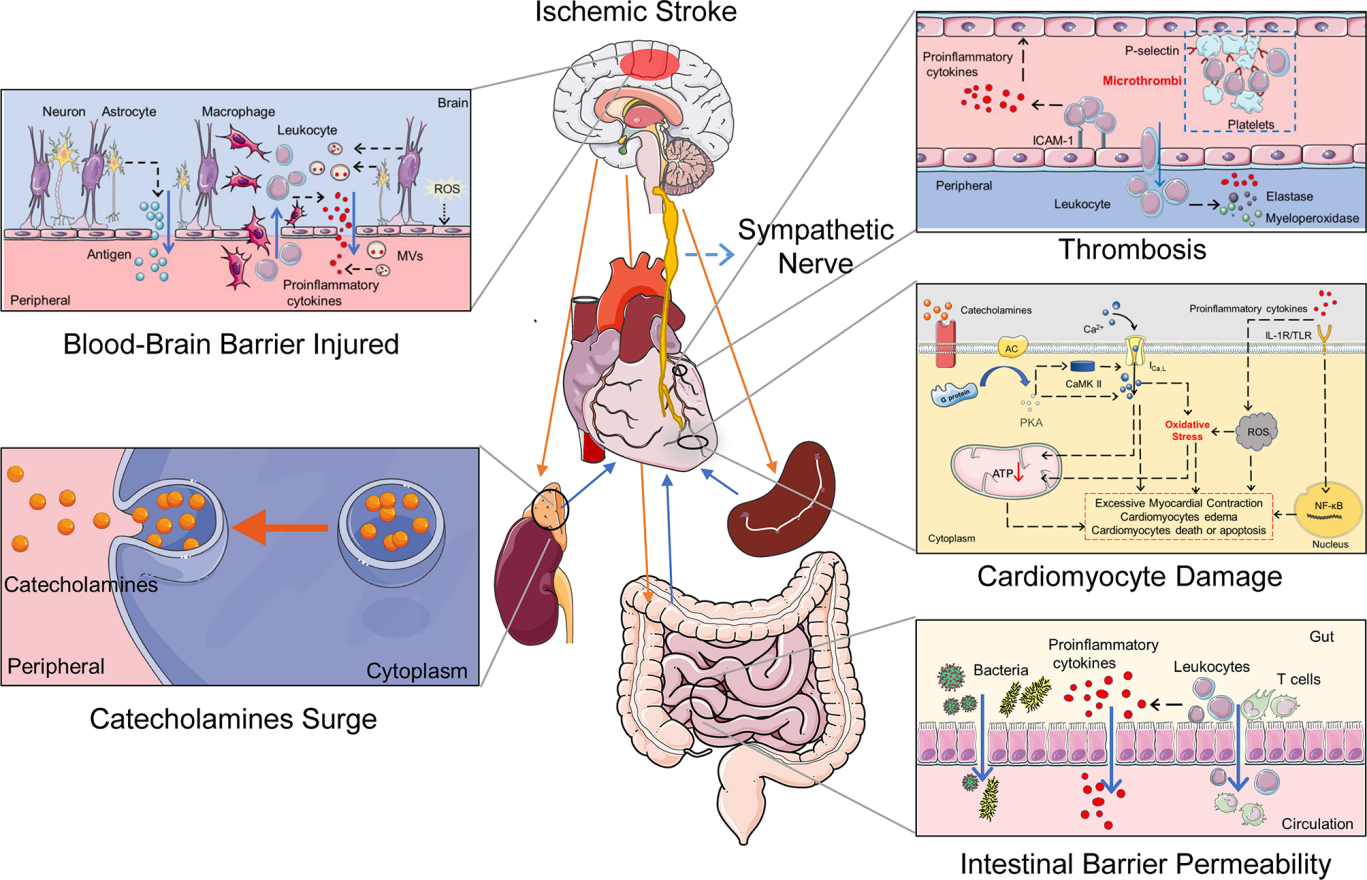
The potential mechanisms of the cerebral-cardiac syndrome in ischemic stroke. Multiple organs and systems work together to mediate cardiac damage after IS. The HPA axis and sympathetic nerve activation after IS induce a catecholamine surge. Catecholamines activate the G protein–AC–cAMP–PKA cascade and increase cytosolic Ca^2+^ in the cardiomyocytes. Intracellular Ca^2+^ overload causes cardiomyocyte damage directly. Simultaneously, sympathetic nerve activation causes gut dysfunction and promotes the transfer of bacteria and proinflammatory cytokines. In addition, IS damages the BBB and neurons, which leaks the inflammatory activators to the peripheral. The spleen releases a variety of immune cells and participates in the activation of systemic inflammation after IS. Increased inflammatory cells and proinflammatory cytokines induce thrombosis and oxidative stress in cardiomyocytes, ultimately leading to cardiac damage. IL-1R, IL-1 receptor; TLR, Toll-like receptor; ROS, reactive oxygen species.

Clinical and experimental studies have explored the potential mechanisms of CCS. However, experimental animal studies provide the possibility to detect the exact pathophysiological mechanisms and related molecular pathways of CCS. The middle cerebral artery occlusion (MCAO) model is widely used for studying IS, which has been shown to cause different types of cardiac damages (11, 49–51). As revealed by experimental animal studies, the possible mechanisms of CCS include insular cortex injury, autonomic imbalance, catecholamine surge, systemic inflammation activation, and the myocardial ionic channel changed after IS (**Table 2**). All of the factors co-contributed to cardiomyocyte pathological damage, microcirculation disorders, and microvascular damage, resulting in arrhythmia, cardiac dysfunction, myocardial infarction, and myocardial fibrosis. Here we discuss the potential pathophysiological mechanisms of CCS (**Figure 1**).

**Table 2:** Cardiac damage in experimental animals after IS.

Animal	Ischemic model	Ischemic area	Cardiac damage	Potential mechanism
Mice	Middle cerebral artery occlusion (MCAO)	Right middle cerebral artery (MCA) area	Cardiac dysfunction, myocardial hypertrophy, and fibrosis	Inflammatory response (52)
				Inflammasome activation (15)
		Left MCA area	Cardiac dysfunction	Catecholamines surge (53)
		Right/left MCA area	Chronic cardiac systolic dysfunction and myocardial fibrosis	Sympathetic overactivity (9)
	Photothrombosis-induced	Right MCA cortex without insular cortex	Cardiac dysfunction, myocardial fibrosis, and hypertrophy	Inflammatory response (11)
		Frontal and parietal cortex	Cardiac dysfunction, myocardial fibrosis, and capillary rarefaction	Inflammatory response and oxidative stress (49)
Rat	MCAO	Right MCA area	Heart rate variability change	Autonomic imbalance (50)
			Cardiac dysfunction and myocardial damage	Oxidative stress (54)
			Prolonged QT and arrhythmia	Ionic channel change (55)
			Cardiac systolic and diastolic function	Ionic channel change (56)
			MAP decline and myocardial damage	Catecholamines surge (57)
			Cardiac myocytolysis	Catecholamines surge (58)
		Right/left MCA area	Myocardial damage and ECG abnormality	Ionic channel change (59)
			ECG changes and myocardial damage	Sympathetic overactivity (60)
	Polystyrene microsphere-induced	Right/left hemisphere	Cardiac dysfunction and increased cardiac vulnerability	Cardioprotective signaling pathway injury (51)
	Endothelin-1 induced	Right/left insular cortex	Endothelial dysfunction and myocardial fibrosis	Inflammatory response (61)
Cat	MCAO	Left MCA area	Myocardial damage	Catecholamines surge (62)
Rhesus macaque	Transient global ischemia	Global cerebral area	Myocardial apoptosis	Inflammatory response (63)
*In vitro*	Oxygen-glucose deprivation	Primary neuronal cells	Reduction in myocardial viability	Cell death signal (64)

#### Autonomic Imbalance Induced by Insular Damage

Insular cortex is important for the central autonomic neural network. Stimulation of the right insular cortex induces significant sympathetic response, while the left insular cortex stimulation enhances the parasympathetic tone (65). Insular cortical lesions commonly occur in the context of IS (66), causing autonomic imbalance and hence leading to cardiac arrhythmia and neurogenic cardiac damage. Up to 88% of patients with insular ischemic damage develop cardiac damage within weeks (22). Of note, cardiac dysfunction and arrhythmia are more severe in patients with right hemisphere insular cortex lesions when compared to those with left insular cortex lesions (1), which may be due to the incontrollable right insular cortex and therefore predisposing to rhythm instability. Cardiomyocyte damage after stroke is characterized by myofiber change to a granular staining pattern, mainly located in the cardiac nerves and the cardiac conduction system region (6, 31). This finding suggests that cardiac autonomic imbalance may damage cardiomyocytes directly. However, another study noted that autonomic dysfunction in patients within 6 months of IS mainly manifests as parasympathetic dysfunctions (67). In brief, autonomic imbalance induced by insular damage after stroke is an important pathogenesis for CCS. However, IS that is unrelated to the insula can also induce cardiac damage (68), suggesting that other pathogenic mechanisms are involved.

#### Catecholamine Surge

Catecholamine surge is the most common hypothesis for CCS. The hypothalamic–pituitary–adrenal (HPA) axis and sympathetic nerve activation are the main sources of catecholamines (69), which are activated after IS (9, 70). Increased catecholamines in the heart and circulation are closely related to the occurrence of arrhythmias and cardiomyocyte damage after IS (53, 57, 58, 60). Briefly, catecholamines activate G protein-coupled receptors, which modulate the G protein–adenyl cyclase (AC)–cyclic adenosine monophosphate (cAMP)–protein kinase A (PKA) cascade in cardiomyocytes (71). PKA can increase cytosolic calcium (Ca^2+^) *via* activating Ca^2+^/calmodulin-dependent protein kinase II (CaMK II) and phosphorylating L-type calcium channels (72). Intracellular Ca^2+^ overload not only provokes cardiac pathological changes such as cardiac myofibrillar degeneration and contraction band necrosis (71) but also causes mitochondrial dysfunction and decreased ATP synthesis, resulting in osmotic cell expansion and ultimately causing cardiomyocyte death or apoptosis (73). On the other hand, catecholamines activate β_3_ adrenal receptors of adipocytes and induce fatty acids releasing into circulation. The free fatty acid accumulation in cardiomyocytes causes energy metabolic disturbance and promotes cardiac inflammation (69). Furthermore, catecholamines act on α_1_ receptors and then produce diacylglycerol and inositol triphosphate, causing coronary vasoconstriction. In addition, the oxidation product of catecholamines, aminochrome, also has cardiomyocyte and cardiovascular toxicity effects (vasoconstriction) (74). Overall, the activated HPA axis and sympathetic nerves induce catecholamine surge after IS and make great contribution to CCS.

#### Immune Response and Systemic Inflammation

The immune response and systemic inflammation after IS are known to exacerbate neurological impairment (75), which could be the responsible source for the subsequent CCS incidence. It has been reported that more than 50% of IS patients experience systemic inflammation within the initial 15 days (76) with a complex process involving immune cells and is driven by multifarious cytokines, proinflammatory chemokines, and sympathetic activity. The blood–brain barrier (BBB) is damaged after cerebral ischemia, followed by subsequent peripheral immune cell infiltration, which worsens BBB destruction afterward (77, 78). The infiltrated microglia and macrophages can release multiple proinflammatory cytokines into periphery circulation, such as IL-6 and IL-1β (79). Damaged neurons, astrocytes, and endothelial cells also produce peroxiredoxin family proteins, brain antigens, and microvesicles (2). These substances are generally confined to the cytoplasm or extracellular spaces in the brain and are released to the general circulation through the damaged BBB and cerebrospinal fluid drainage pathways, promoting immune response and systemic inflammation after IS (80). In the peripheral organs, spleen and gut are the major sources to regulate systemic inflammation after IS. It is undisputed that immune cells in the spleen are activated by IS (81). Meanwhile, sympathetic activation and catecholamine surge cause mesenteric vasoconstriction and intestinal paralysis, exacerbate intestinal ischemia, and increase gut–blood barrier disorder and proportional gut permeability, which promote intestinal bacterial and immune cell translocation from the gut to the peripheral organs (82). Furthermore, microbiome imbalance and the increased immune cells provoke severe systemic inflammation after IS (2).

Overactivation of the systemic inflammation causes cardiac pathological damage directly. As indicated, the activated immune response and systemic inflammation increase proinflammatory cytokines, which target the IL-1 receptor, the Toll-like receptor, or the transcription factor nuclear factor-kappa B pathway to promote reactive oxygen species (ROS) production and to reduce the synthesis and bioavailability of nitric oxide (83). The inflammatory microenvironment also favors leukocyte activation and adhesion to the vasculature. Leukocyte-endothelial cell adhesion and platelet aggregation are mediating microthrombi and microvascular dysfunction, cardiomyocyte dysfunction, edema, and eventually cell death (84). Importantly, the increased monocytes and macrophages have been found in cardiac tissue after IS, which boost cardiac inflammation and hence promotes cardiac damage (49, 63). Inhibiting inflammatory responses have been proven as an effective way to attenuate cardiac damage in CCS (11, 49, 52). All in all, increased inflammatory cytokines and immune cells after IS lead to cardiac pathological damage, which participates in the pathological process of CCS.

## Diabetic Status Exacerbates Cerebral-Cardiac Syndrome

### Clinical Evidence

Diabetes increases the incidence of cardiac complications after IS, which manifest as myocardial infarction, congestive heart failure (85), arrhythmia, cardiac arrest (16), and exacerbate mortality and worse neural prognosis in the post-stroke state (15, 49, 86). Burkot et al. have reported that heart failure occurrence rate after IS is 22.3% in diabetic patients and 15.1% without diabetes (10). The level of myocardial enzyme from damaged myocardium are higher in diabetic IS patients, such as cTnI and hs-cTnT, which have strongly associated with atrial fibrillation (48). Furthermore, compared to the non-diabetic patients, the incidence of myocardial infarction was 3.5 times higher in diabetic patients in the post-stroke state (87). A recent study showed that canagliflozin (an anti-hyperglycemic drug treats diabetes) can significantly reduce the incidence of myocardial infarction and cardiovascular death after stroke (85). Another case report has also suggested that insulin therapy is beneficial for stroke patients with complicated diabetes who suffered from neurogenic stunned myocardium (88). Together, these studies suggest that a potential connection may exist between diabetes and CCS.

### Potential Pathogenesis

**Figure 2:**
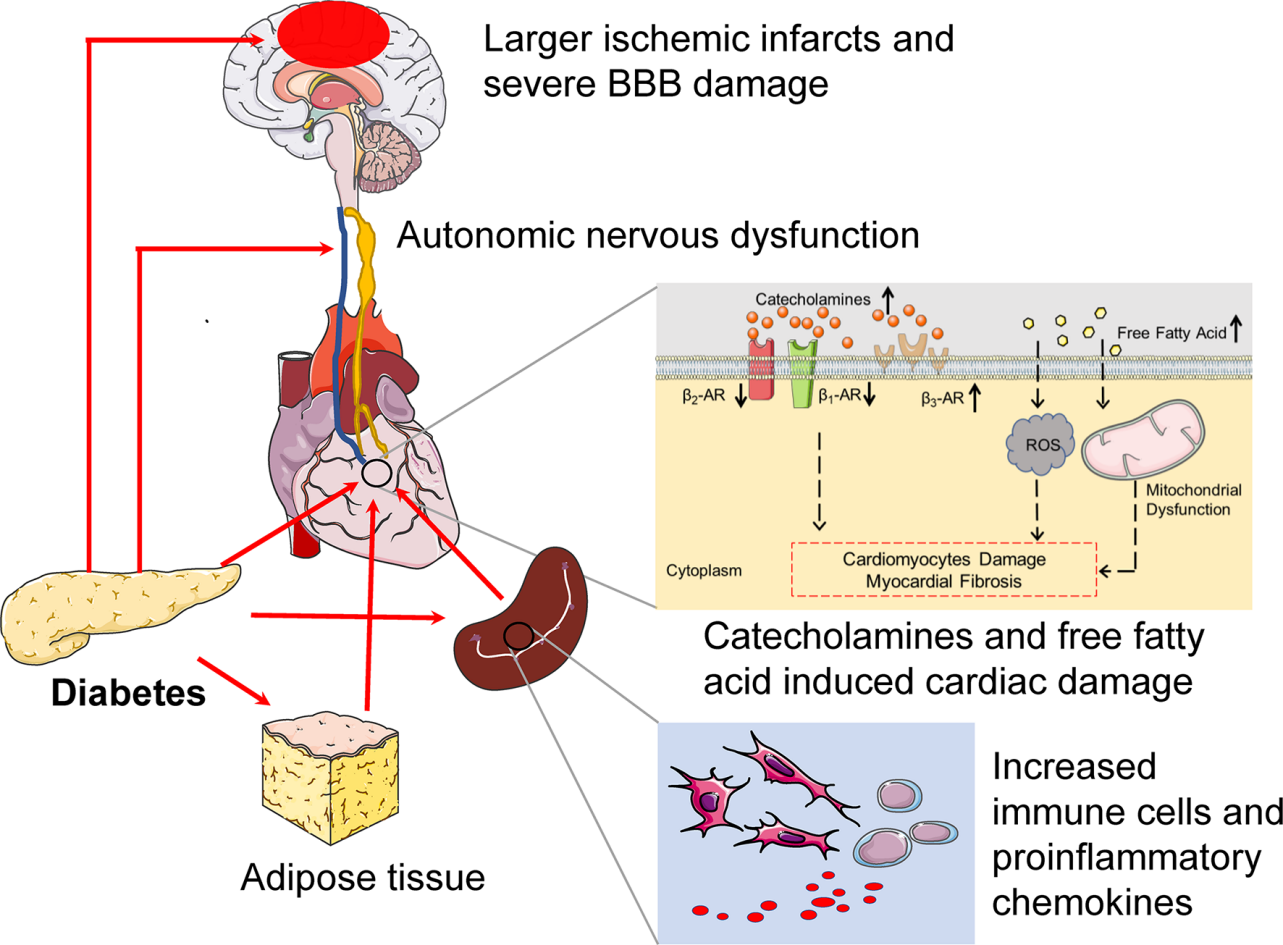
The potential pathogenesis of diabetes exacerbates cerebral-cardiac syndrome. In pre-stroke states, diabetes induces preexisting injury both of the brain and the heart. CAN is a common complication in diabetes, which is characterized by parasympathetic denervation and overactivated sympathetic tone. Furthermore, the expression of β_1_-AR and β_2_-AR decreased, while the β3-AR increased in the diabetic heart. Diabetes also provides a low-grade systemic inflammatory environment, which manifests as increased immune cells and proinflammatory cytokine levels. All of these provide a suitable condition for CCS. In the context of diabetes, the larger ischemic infract and severe BBB damage can be detected during the IS onset, which provoke severe systemic inflammation and cause severe cardiac inflammatory damage. Catecholamines surge also increase after IS in diabetes. Catecholamines surge not only damage cardiomyocyte directly but also act on adipose tissue, which increases free fatty acids. The boosted free fatty acids induce ROS production and mitochondrial dysfunction, which promote cardiac damage. Above all, diabetes induces preexisting cardiac damage and increases heart vulnerability, and cultivates a “primed” inflammatory microenvironment before IS and severe systemic inflammation and catecholamines surge after IS, hence exacerbating CCS. CAN, cardiac autonomic neuropathy.

The severity, duration, and clinical manifestation of diabetes should be considered when facilitating the CCS, and there are a few literatures that focus on it. The exact mechanisms contributing to CCS in the context of diabetes are obscure. However, available evidences are emerging (15, 49). Firstly, diabetes increased the vulnerability and susceptibility of both the brain and the heart in the pre-stroke stage. Secondly, diabetes is known to induce systemic inflammation and to trigger oxidative and hyperosmolar stress, which lead to more severe cardiac inflammatory damage in the diabetic stroke status. Herein we characterize the potential pathogenesis to show how diabetes affects CCS (**Figure 2**).

#### Autonomic Neuropathy in Diabetes

The autonomic imbalance is an important mechanism of CCS. Diabetic stroke leads to larger ischemic infarcts and more severe neurological deficits. Severe cerebral damage may be the basis for exacerbating CCS (19, 89). Diabetic autonomic neuropathy is common in diabetic patients, and cardiac autonomic neuropathy (CAN) is one of the common subtypes (90). CAN is characterized by parasympathetic denervation and augmented sympathetic tone (91). A stronger sympathetic tone can cause severe cardiac damage after the IS onset (9).

#### Boosted Catecholamine Surge and β-Adrenergic Receptors Disorder in Diabetes

Diabetes provokes cardiac sympathetic nerve excitation, metabolic disorder, and subsequent catecholamine surge (92). Catecholamine surge can induce cardiac pathological damage directly (93). In addition, catecholamine surge acts on adipose tissue and hence increases free fatty acids after IS. Myocardial insulin resistance leads to a reduction in regional glucose utilization, and the myocardial energy supply balance is shifted to free fatty acid metabolism in the context of diabetes (94). Hence, the demand for free fatty acids in the myocardium may increase in diabetic IS. However, excessive free fatty acids can induce cardiomyocyte damage, myocardial fibrosis, and cardiac remodeling *via* increasing myocardial oxygen consumption, ROS production, and myocardial mitochondrial dysfunction (95). The cardiac β-adrenergic receptors (β-ARs) are the main receptors of catecholamine. Diabetic status alters the expression and responsiveness of β-AR of the myocardium, manifesting as β_1_-AR and β_2_-AR decreased while β_3_-AR increased (96). It is believed that β_2_-AR activation provides cardioprotection. However, β_2_-AR displayed a reduced reactivity of adrenergic stimulation in the diabetic heart, which caused the exercise capability damage and impaired cardiac contractile in mice (97). Together, the superimposed catecholamine surge combined with the β-ARs disorder may be responsible for severe CCS in diabetes.

#### Diabetes Exacerbates Cardiac Inflammatory Damage

As immune response and systemic inflammation are important triggers for CCS, it is rational that other risk factors that share semblable inflammatory mechanism also potentiate the progression of CCS. Diabetes indicates a preexisting chronic low-grade systemic inflammatory disease that manifests as elevated serum leukocyte counts and C-reactive protein, adiponectin, and proinflammatory cytokine levels (98). Furthermore, diabetic patients suffer from severe systemic inflammation after IS, manifesting as increased activation of circulating neutrophils and monocytes (99). In diabetes, macrophage mobilization and infiltration have been observed in the heart even in the absence of stroke (100), and macrophage infiltration is more obvious after IS (15). Increased macrophages and neutrophils form an inflammatory activation loop and cause severe inflammatory damage (101). Diabetes affects BBB integrity and permeability, which may increase the leakage of multiple inflammatory factors from the brain after stroke (102). These findings strengthen the hypothesis that diabetes establishes a “primed” suitable environment before IS and drives severe systemic inflammation after IS, which may exacerbate cardiac inflammatory damage and induce CCS. Anti-inflammatory therapy *via* reducing the inflammation and macrophage infiltration in the heart after IS may be a potential treatment for diabetic CCS (15, 49). Therefore, excessive systemic inflammation is an indispensable pathogenesis in severe CCS in the context of diabetes.

## NLRP3 Inflammasome in Diabetic Cerebral-Cardiac Syndrome

Inflammation is no doubt a direction that needed further exploration in CCS, particularly for diabetes state. Recently, the NLRP3 inflammasome has been broadly studied of its close relationship with inflammation diseases (103). Importantly, NLRP3 inflammasome activation plays an indispensable role in diabetes and diabetic complications (104, 105). However, it is unclear whether diabetic CCS relies on NLRP3 inflammasome activation. How does NLRP3 inflammasome participate in the onset of diabetic CCS? Which cell types in the heart response to the activated NLRP3 inflammasome after IS and how does it lead to cardiac damage? The following sections will focus on the recent findings that refer to the above questions.

### NLRP3 Inflammasome Activation Induced Cardiac Damage in Diabetic Cerebral-Cardiac Syndrome

NLRP3 is highly expressed and activated in immune cells (103). The NLRP3 inflammasome contains NLRP3, the apoptosis-associated speck-like protein containing and the effector cysteine protease caspase-1 (105). Various danger signals activate the NLRP3 inflammasome by pathogen- and damage-associated molecular patterns, such as high glycemic environments and bacterial and viral nucleic acids (13). The activated NLRP3 inflammasome further promotes the maturation and release of IL-1β and IL-18, as extensively described in a recent review (13).

Of note, NLRP3 inflammasome activation is an important pathogenic mechanism of diabetes and diabetic complications (104, 105). NLRP3 inflammasome activation induces insulin resistance and impairs pancreatic β-cells, participating in the development of diabetes (14). NLRP3 inflammasome-IL-1β secretion in cardiac macrophage induces a decrease in potassium current and an increase in calcium sparks in cardiomyocytes, promoting the development of diabetes-induced arrhythmia (106). Our previous study has suggested that NLRP3 inflammasome activation increased in the myocardium after IS in a diabetic mouse model (15), indicating that the NLRP3 inflammasome may play an important role in diabetic CCS. NLRP3 inflammasome can be activated in the heart after IS. Firstly, proinflammatory cytokines, microvesicles, and antigens are leaked from the ischemic brain (2). Bacteria, immune cells, and other toxic substances such as the microbial metabolite trimethylamine translocate after IS-induced gut–blood barrier disorder (2), which can impact the myocardium and activate the cardiac NLRP3 inflammasome. Secondly, catecholamine surges causes intracellular CaMK II activation, Ca^2+^ overload, and ROS production, which are directly or indirectly involved in the activation of NLRP3 inflammasome (107). Also, IS-induced mitochondrial dysfunction in cardiomyocytes causes the loss of mitochondrial membrane potential and release of mitochondrial DNA into the cytosol, which further activates NLRP3 inflammasome (54). Furthermore, hyperglycemia can also activate NLRP3 inflammasome in the myocardium (108). Overall, the cardiac NLRP3 inflammasome may be more sensitive to be activated in diabetic CCS. Sequentially, the activated NLRP3 inflammasome exacerbates cardiac inflammatory damage *via* mediating the maturation of IL-1β and IL-18 (109).

A previous study has suggested that NLRP3 inflammasome is mainly activated in cardiac monocyte-derived macrophages under the pathological state (110). However, NLRP3 inflammasome is expressed in various cardiac cell types including cardiomyocytes, fibroblasts, microvascular endothelial cells, and cardiac macrophages (109). NLRP3 inflammasome activation in different cardiac cells may play a different role in diabetic CCS. NLRP3 inflammasome activation in cardiomyocytes promotes abnormal sarcoplasmic reticulum Ca^2+^ release, causing ectopic firing and augmented K^+^ currents that abbreviate electrical remodeling and promote the development of atrial fibrillation (111). In myocardial fibroblasts, NLRP3 inflammasome activation regulates mitochondrial ROS production, which eventually leads to the expression of profibrotic genes (such as collagen one and α-smooth muscle actin) and then provokes myocardial fibrosis and cardiac remodeling (112). NLRP3 inflammasome activation also enhances macrophage recruitment, which is involved in the progression of cardiac inflammation and the formation of plaques and destabilizes the plaque, which increased the incidence of myocardial infarction (113, 114). However, NLRP3 inflammasome activation not only increases the number of macrophages but also affects the macrophage polarization state. Generally, macrophage polarization can be divided into the proinflammatory M1 type and the anti-inflammatory M2 type. M1 macrophages promote cardiac inflammation, cardiac fibrosis, and cardiac dysfunction, while M2 macrophages reduce cardiac inflammation and remodeling (115). NLRP3 inflammasome activation provokes macrophage to M1 type and hence exacerbates cardiac inflammatory injury (116). Inhibiting the NLRP3 inflammasome activation can promote macrophage to M2 polarization, which attenuates post-infarct cardiac dysfunction (117).

**Figure 3:**
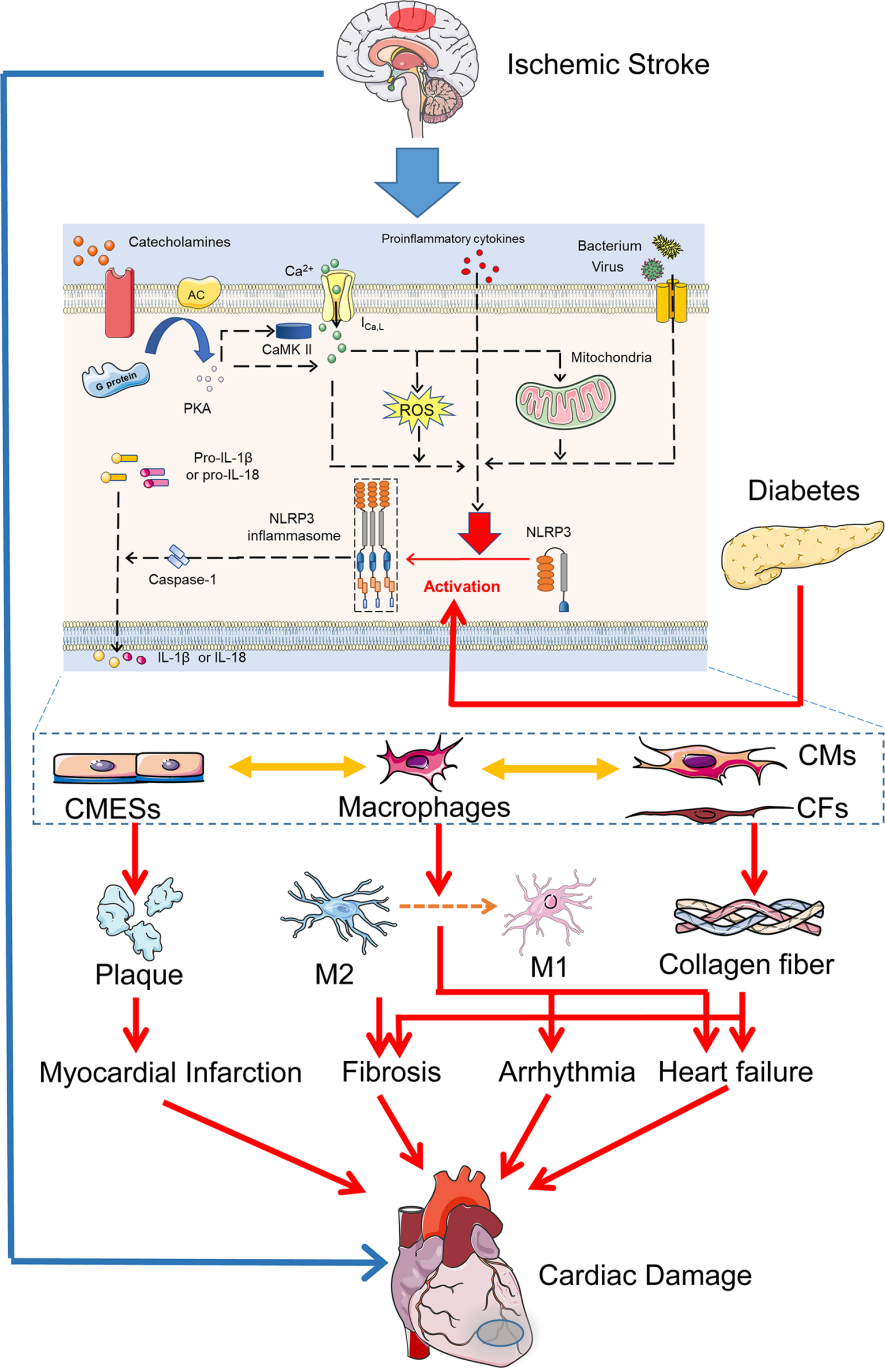
NLRP3 inflammasome activation in diabetic cerebral-cardiac syndrome. Increased catecholamines, proinflammatory cytokines, and bacteria after IS induce intracellular Ca^2+^ overload, mitochondrial dysfunction, ROS production, and activation of CaMKII in cardiac cells. These activators and hyperglycemia in diabetes directly activate the NLRP3 inflammasome in various cardiac cell types. NLRP3 inflammasome activation in CMESs induces platelet and macrophage aggregation. In cardiac macrophages, NLRP3 inflammasome activation promotes M1 macrophage polarization and provokes the cardiac inflammation damage. In CMs and CFs, NLRP3 inflammasome activation increases the expression of profibrotic genes and promotes myocardial fibrosis. Overall, IS and diabetes coactivate NLRP3 inflammasome in various cardiac cells, which ultimately leads to arrhythmia, myocardial fibrosis, myocardial infarction, and heart failure, resulting in diverse cardiac damage. CMESs, cardiac microvascular endothelial cells; CMs, cardiomyocytes; CFs, cardiac fibroblasts.

In summary, diabetic stroke induces NLRP3 inflammasome activation *via* various mechanisms in cardiac macrophages, microvascular endothelial cells, cardiomyocytes, and cardiac fibroblasts, which increase cardiac inflammation, recruit macrophage infiltration, and induce macrophage polarization. Excessive inflammation ultimately leads to cardiomyocyte necrosis, contraction band necrosis, plaque rupture, endothelial dysfunction, and the activation of myofibroblast (3, 118, 119), resulting in arrhythmia, myocardial fibrosis, myocardial infarction, and heart failure (**Figure 3**).

## Conclusions and Future Direction

Conclusively, we summarize the features and the responsible mechanisms of CCS in the review. CCS refers to the cardiac damage after IS that has various clinical manifestations, including arrhythmia, myocardial damage, and heart failure. Cardiac complications predict the development and prognosis of IS in turn. In addition, we summarize the responsible mechanisms in CCS, which include insular cortex injury, autonomic imbalance, catecholamine surge, immune responses, and systemic inflammation after IS. Furthermore, we try to indicate the role of diabetes in CCS and the link of NLRP3 inflammasome between diabetes and CCS. Of note, diabetes is a risk factor in CCS (15). On one hand, diabetes induces preexisting injury in the heart before CCS onset. The preexisting injury increases the vulnerability and susceptibility of cardiac damage after IS and provides a suitable environment for CCS. On the other hand, boosted catecholamine surge and severe inflammatory response provoke cardiac pathological changes and aggravate CCS after the IS onset in diabetes. As the key element of inflammation, NLRP3 inflammasome plays an essential role in inflammation diseases (103). Therefore, we further focus on the role of NLRP3 inflammasome in diabetic CSS. We characterize the ways of NLRP3 inflammasome activation and the subsequent cardiac pathological damage in CCS combined with diabetes and propose that NLRP3 inflammasome-mediated inflammation may be a potential target in diabetic CCS. However, the other pathogenesis of diabetic CCS is also worthy of further investigation. Overall, it is important to strengthen post-stroke cardiac monitoring in the clinic, especially in diabetic patients, which may reduce the mortality associated with IS.

## Author Contributions

H-FZ, F-XL, H-BL, W-BL, Z-JY, and S-YX contributed to the review including the designing and planning. H-BL, F-XL, and J-YZ wrote the manuscript. W-BL and H-FZ contributed to critically revising the work. All authors read and approved the final manuscript.

## Funding

This work was supported by grant 81771232, 82070526 (to H-FZ) and 81974192 (to F-XL) from the National Natural Science Foundation of China and grant 2019A1515010654, 2021A1515011652 (to H-FZ) from the Natural Science Foundation of Guangdong Province, China.

## Conflict of Interest

The authors declare that the research was conducted in the absence of any commercial or financial relationships that could be construed as a potential conflict of interest.

## Publisher’s Note

All claims expressed in this article are solely those of the authors and do not necessarily represent those of their affiliated organizations, or those of the publisher, the editors and the reviewers. Any product that may be evaluated in this article, or claim that may be made by its manufacturer, is not guaranteed or endorsed by the publisher.

## Abbreviations

CCS, Cerebral-Cardiac Syndrome; IS, Ischemic Stroke; ECG, Electrocardiograph; cTnI, Cardiac Troponin I; cTnT, Cardiac Troponin T; hs-cTnT, High-Sensitive Cardiac Troponin T; LVSD, Left Ventricular Systolic Dysfunction; HPA, Hypothalamus–Pituitary–Adrenal; PKA, Protein Kinase A; BBB, Blood–Brain Barrier; ROS, Reactive Oxygen Species; IL, Interleukin; CAN, Cardiac Autonomic Neuropathy; NLRP3, NOD-like receptor pyrin domain containing 3.
